# Comparison of glucose measurement on dried blood spots versus plasma samples in pregnant women with and without anemia

**DOI:** 10.20945/2359-3997000000229

**Published:** 2020-03-30

**Authors:** Ana Lígia Soares Matos, Jessica Pronestino de Lima Moreira, Ronir Raggio Luiz, Evelise Pochmann da Silva, Melanie Rodacki, Juan Fidel Bencomo Gómez, Lenita Zajdenverg

**Affiliations:** 1 Maternidade Escola Universidade Federal do Rio de Janeiro Rio de Janeiro RJ Brasil Maternidade Escola, Universidade Federal do Rio de Janeiro (UFRJ), Rio de Janeiro, RJ, Brasil; 2 Instituto de Estudos de Saúde Coletiva Universidade Federal do Rio de Janeiro Rio de Janeiro RJ Brasil Instituto de Estudos de Saúde Coletiva (IESC), Universidade Federal do Rio de Janeiro (UFRJ), Rio de Janeiro, RJ, Brasil; 3 Departamento de Medicina Interna Unidade de Diabetes e Nutrologia Universidade Federal do Rio de Janeiro Rio de Janeiro RJ Brasil Departamento de Medicina Interna, Unidade de Diabetes e Nutrologia, Universidade Federal do Rio de Janeiro (UFRJ), Rio de Janeiro, RJ, Brasil; 4 Instituto Vital Brasil Niterói RJ Brasil Instituto Vital Brasil, Niterói, RJ, Brasil

**Keywords:** Glucose, gestational diabetes mellitus, capillary blood in DBS, hematocrit

## Abstract

**Objective:**

Compare the concordance degree between plasma glucose and glucose measurements on Dried Blood Spots (DBS) during pregnancy.

**Subjects and methods:**

Glucose measurement was performed in pregnant women after a fast of 8-12 hours. Venous blood was collected with sodium fluoride, the plasma was separated, and glucose measured by the enzymatic oxidase glucose method. Capillary blood samples were collected and analyzed by DBS. For statistics, the paired Student’s t test, interclass correlation coefficient (ICC), graphic approach of Altman and Bland, and survival – concordance plot were used.

**Results:**

307 pregnant women were evaluated, 88.6% without diabetes and 11.4% with previous diabetes. The glucose ranged from 66 to 190 mg/dL [3.66 to 10.55 mmol/L] in plasma and from 53 to 166 mg/dL [2.94 to 9.21 mmol/L] in DBS. The glucose average values were 88.1 ± 12 mg/dL [4.98 ± 0.67 mmol/L] in plasma and 89.2 ±11,5 mg/dL, [4.95 ± 0.64 mmol/L] in DBS – p-value = 0.084. The ICC value was moderate (0.510), and Pearson’s correlation coefficient was r = 0.507 p < 0.001. Altman and Bland’s graph showed that difference between the values obtained by both methods is -24.62 to 22.3 mg/dL [-1.37 to 1.24 mmol/L]. Significant fixed bias (-1,16 average difference) and proportional bias (r = 0.056; p = 0.33) were not observed. Anemia was associated with differences between plasma glucose and DBS measurements (p = 0.031).

**Conclusion:**

Capillary glucose in DBS correlates with plasma glucose; however, the methods do not present good concordance. The presence of anemia worsens this result.

## INTRODUCTION

Gestational diabetes mellitus (GDM) is defined by the presence of carbohydrate intolerance that starts during pregnancy and results in hyperglycemia of variable degree (1). It is associated with an increased risk of complications for the mother and the child during pregnancy and at birth (2,3). Women with GDM diagnosis have an increased risk of developing type 2 diabetes throughout life (4).

With the spread of diabetes worldwide, there is an urgent need to develop effective strategies to identify populations at risk since it is possible to prevent the development of diabetes (5) through changes in lifestyle or pharmacological intervention (4,6).

There are practical restrictions for GDM screening with plasma glucose for low-income populations. Collection and preparation of blood samples in tubes requires laboratory infrastructure for samples separation, transport, storage, and processing. Dried Blood Spots (DBS) have been used for the analysis of human blood for over 40 years. It began in 1960 for the screening of congenital metabolic and endocrine diseases, such as phenylketonuria and hypothyroidism (7). More recently, it has been used as DNA source for screening genetic abnormalities in newborns, such as cystic fibrosis and hemoglobinopathies. A recent meta-analysis of seventeen heterogeneous studies demonstrated that HbA1c results from DBS were correlated to those obtained through venipuncture. Despite this we did not find consensus or recommendation to use the method for diabetes screening (8).

DBS collected through finger puncturing represents a minimally invasive alternative to venipuncture that facilitates the collection of blood samples in naturalistic, field-based research settings. Blood collection through the DBS technique is simple, offers many advantages, including minimal sample collection volume, reduced costs associated with shipping and storage, it does not require centrifugation to separate the samples and it is less invasive than collecting venous samples (9,10). Moreover, many analytes are stable at room temperature for up to one week (11). Epidemiological studies on diabetes and other metabolic diseases rely on the difficulty of collecting, storing and transporting samples to be processed. These studies require a method that is effective and simple to apply (12). DBS are robust for shipping, they can be stored at room temperature, and are able to be sent by mail without the need of special precautions (13). The technology to quantify glucose in DBS could make screening easier, which would help to detect diabetes in high-risk populations with limited financial resources. Samples of whole blood in DBS might be an option for the successful screening of populations in remote areas where the need of processing blood for laboratory analysis could be a major issue (9,14,15). Capillary samples evaluated by point-of-care (POC) glucose testing methodology for the diagnosis of GDM may be promising (16). However, accuracy is rarely observed in the glucose measurements by the POC method (17). Accuracy of the POC diagnostic criteria may also depend on frequent updates in the glucometers used in different healthcare settings. Because of the imprecision and variability of the glucose meters, this method should not be used to diagnose diabetes and has limited value in screening (18).

In order to confirm reproducibility of DBS, the European Bioanalysis Forum (EBF) has discussed in detail the currently used DBS methodology. The recommendations provided reflect on (potential) differences and point out special considerations needed for validation and sample analysis of DBS compared with traditional liquid assays. The studies in which the samples are analyzed with validated DBS methods need special acceptance criteria. The scope of the questions included the sampling, sample handling, shipment and storage phases (19).

Results from DBS samples represent the concentration of an analyte in whole blood, which, by definition, will differ from results determined in plasma. Except for newborn screening, DBS samples are not the clinical standard (20). The quantitative implementation into clinical routine has been developed in the last four to five years. The analytical validation of a quantitative DBS method should be conducted first according to the FDA and EMA guidelines (21).

Special populations, such as patients with physiological conditions or under medical treatment that affects the hematocrit (Hct), may require additional validation (22). Hct is currently identified as the single most important parameter influencing the spread of blood on DBS cards, which could impact the validity of the results generated by DBS methods, affecting the spot formation, spot size, drying time, homogeneity and, ultimately, the robustness and reproducibility of the assays (22,23). The Hct has considerable effect on blood viscosity. It can have a direct influence on the accuracy and precision of the analysis in bioanalytical essays using DBS (24). Anemia falsely increases and polycythemia falsely reduces glucose levels in capillary blood (25), which could be a limitation for the use of DBS in the screening of GDM.

The purpose of this study is to compare the degree of concordance between the plasma glucose measurement (reference standard) and the glucose value when analyzing DBS samples (using ELISA type microplate) during pregnancy, checking the interference of hematocrit.

## SUBJECTS AND METHODS

The study was conducted in the Maternidade Escola da Universidade Federal do Rio de Janeiro/Brazil, in partnership with Instituto Vital Brazil, Niterói, Rio de Janeiro, Brazil. The project was approved by the local ethic committee.

We included all pregnant women who volunteered to perform the plasma glucose test in the prenatal routine of the institution. Data collection was performed between May and August 2011. Those under 18 years old and those who did not sign the consent form were excluded.

In order to achieve results with necessary reliability, the reactants and standards were carefully prepared. Statistical assays following standardization, accuracy, sensibility, and specificity analyses for validation of the technique and to define results reproducibility were applied to the initial technical steps to quantify glucose in the blood collected in DBS. To assure the quality and the reliability of the method provided, two standards and two levels of internal quality control were used such that the assays are performed in duplicate after the elaboration of the work map. The obtained variation coefficient of the method was: 1,3% intra-assay and 2,91% intra-assay.

For the DBS glucose measurement, filter paper type BSM0705 from Albert - Hahnemuehle S.L. (Germany), made of 100% cotton and featuring five numbered zones, each one with 2 cm in diameter, was used.

Venous blood (3 mL) was collected in a closed blood collection system, containing sodium fluoride (BD Vacuutainer^®^ Sodium Fluoride Potassium Oxalate 5 mg/4 mg, Doles Reag. Equip. for Laboratories Ltda., Brazil). After that, plasma separation was performed in a centrifuge at room temperature, Relative Centrifugal Force (g) = 2,195.2 g. The glucose was determined with the Automatic Analyzer A15 unit (Biosystem^®^, Spain) by the Enzymatic oxidase method – Reference Standard.

Simultaneously, capillary blood samples were collected through finger puncturing with a lancet. After digital asepsis, the first blood drop was ignored and the five circles of the filter paper were filled up according to the standard protocols for collection, storage, and transportation of blood on filter paper (20,21,26-28). These samples were analyzed by the DBS method (using ELISA type microplate) in Biomarc Laboratory (Niterói, Brazil)

Two glucose samples were prepared using human blood from volunteer donors without diabetes. With the absorbance results of these control samples, the cotangent was calculated to determine the concentration values of the samples.

The measurement of plasma and capillary glucose levels was performed by two different technicians and the results were sent to the central analyzer for comparative study.

Blood counts were performed by the ABX Pentra C + Horiba Medical^®^ device (France). The blood count was performed in 236 patients. After calibration, control samples and test samples were analyzed. The results included hematocrit, hemoglobin, platelets and differential leukocyte counts. Hemoglobin (Hb) is the iron-containing oxygen-transport protein in the red blood cells (erythrocytes) of almost all vertebrates. The hematocrit (or packed cell volume) is the percentage of volume occupied by red blood cells compared to the total blood volume. The Hct result was used in this study to identify anemia. It also can be obtained using a centrifuge microhematocrit, a simple and cost-saving approach, which is a portable and practical equipment for use in field work. It requires a simple and short training period to operate, as well as a stable power source. It requires about 0.5 µL of blood, equivalent to a drop, extracted in a capillary tube, closed at one end and centrifuged (29).

The hematocrit below 33% is an acceptable indicator for evaluation and identification of anemic patients with hemoglobin in place. There is a standard conversion between the two measures (Hb = Ht/3) which is commonly used to define cut-off points for estimating the prevalence of anemia. Health services have been using this indicator for anemia screening, since it is less expensive and more practical, but reliability studies are needed (29). The samples were categorized, according to the protocol of the World Health Organization (WHO, 2001) (30), as anemic (Hct < 0.33 g/%) and non-anemic (Hct ≥ 0.33 g/%) pregnant women using conventional conversion factors (29).

In pregnant women, anemia is defined when Hb is below 11 g/dL. Conventional conversion factors are: 110 g/L hemoglobin = 0.33 g/% hematocrit. These are sea level values for hemoglobin and hematocrit corresponding to anemia.

DBS discs were cut into the puncher in 3 mm sizes, ensuring that samples were properly collected. Each DBS disc corresponds to 5 µL of whole blood, equivalent to one drop (29), and it was placed with a needle in the well of the ELISA plate as previously identified.

Using a multichannel pipette, 75 µL of AM2 (Alcohol/Methanol) solution (eluate) was distributed into each well of the plate containing the DBS. The plate was immediately sealed with covers suited for ELISA type plates to prevent evaporation, and then the plate was incubated for 120 minutes at 37 ºC in a shaker with an average speed of 200 rpm for the glucose extraction.

Another ELISA type plate was employed and 30 µL of elution were dispensed on the plate, gently homogenizing it, and following the samples order of the previous plate. The quantity suctioned by each pipette was 30 µL, the eluate was poured at the bottom of the plate, and each column was quickly covered to prevent evaporation of the sample.

The standard sample was repeated at the beginning and at the end of the plate to ensure its reproducibility. A1 and A2 holes were used as BLANK, and C1 and D1 as STANDARDS of known concentration for the cotangent calculation. In all wells, 150 µL of the reactive enzyme glucose solution (glucose - oxidase ) was added and gently homogenized. After this phase, where the enzymatic reaction occurs, the plate was incubated at 37 ^o^C for 20 minutes to complete the reaction. Its absorbance was read on a 492 nm filter ELISA microplate reader and its concentration was calculated using the cotangent obtained between absorbance and concentration standards. The absorbance values obtained by the capillary blood samples in DBS were noted in an Microsoft^®^ Office Excel spreadsheet and then the standard value and program calculation tools were used to calculate the actual value of glucose using the following formula: 
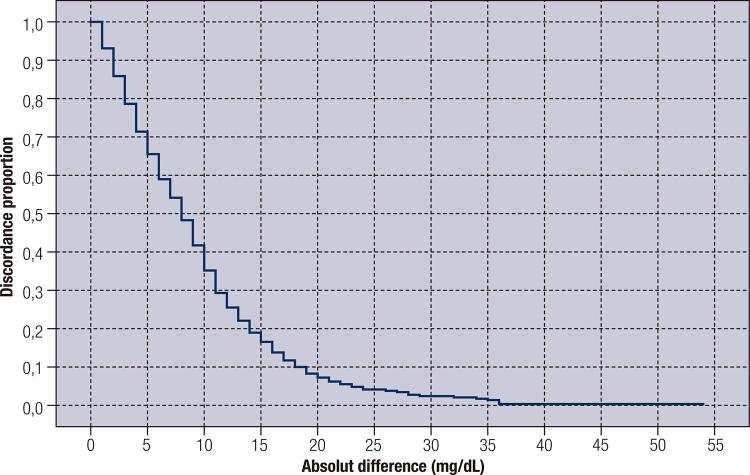
(CV = Variation Coefficient, DP = Standard Deviation, X = Average Group and Values).

The optical density or absorbance (DO) values were obtained in an ELISA PR2100 spectrophotometer. The studied analyte concentration was calculated using the co-tangent of the calibration curve, by placing the glucose concentrations in the abscissa axis as an independent variable, and the absorbance values in the ordinate axis as dependent variables. The Revelation Program, designed for this procedure, selects the Glucose technique and performs the plate reading. Afterwards, the computer performs analytical calculations and connects its values to the pre-analytical processing data previously stored in the registration process.

An ELISA PR2100 microplate reader (Sanofi Pasteur^®^, France), was used coupled to the computer to provide automated processing of values calculations and data storage.

A stackable sterile microtiter ELISA plate provided with a flat bottom, 96 wells, a lid, and alphanumerical identification with lateral labelling area was used.

One glucose measurement was performed in plasma samples and two different measurements were performed in DBS. For the comparison study, the average of two determinations in DBS was used. A cut-off point ≥ 92 mg/dL [5.11 mmol/L] (IADPSG) (3) was assumed as the cut-off point for the classification of altered fast glucose in pregnant patients.

For statistical analysis, the SPSS (18.0 version) program was used. The paired Student’s *t* test was used to compare the average glucose values obtained by the two methods, the graphical approach of Altman and Bland (31) to evaluate the concordance as well as the survival-concordance plot to assess the limits of concordance between the two methods.

## RESULTS

A total of 307 pregnant women aged between 18 and 48 years old (an average of 28.7 years) were evaluated. They varied gestational age between the 5th to the 42nd gestation week; 31.5% were between the 5th and the 12th weeks, 35% between the 13th and the 24th weeks and 30.3% over the 25th week; and in 3.2% of the cases the gestational age was unknown. Out of the total number of participants, 272 (88.6%) did not have previous diabetes, while 35 (11.4%) had previous diabetes.

The glucose in plasma and in DBS ranged from 66 to 190 mg/dL [3.66 to 10.55 mmol/L] and from 53 to 166 mg/dL [2.94 to 9.21 mmol/L], respectively. The glucose average values in samples collected in plasma and in DBS were, respectively, 88.1 ± 12 mg/dL [4.98 ± 0.67 mmol/L] and 89.2 ± 11,5 mg/dL, [4.95 ± 0.64 mmol/L] *p*-value = 0.084. The ICC value was moderate, 0.510 (0.402-0.604), and Pearson’s correlation coefficient was r = 0.507, *p *< 0.001. The average difference between plasma glucose and DBS glucose was -1.16 mg/dL [0.06 mmol/L] (Table 1).

**Table 1:** Descriptive statistics of plasma and DBS glucose (mg/dL) in pregnant women – N = 307 Glucose (mg/dL)

Descriptive statistics	Glucose in Plasma (A)	Glucose in Filter Paper (B)	Difference (A-B)
Average	88.1	89.2	-1.16
Standard			
deviation	12.1	11.5	11.7
Minimum	66	53	-36
First quartile	81	82	-9
Median	86	87	-1
Third quartile	93	95	6
Maximum	190	166	54
P-value of paired-test	0.084		
ICC			**0.510**

ICC: interclass correlation coefficient.

**Figure 1:**
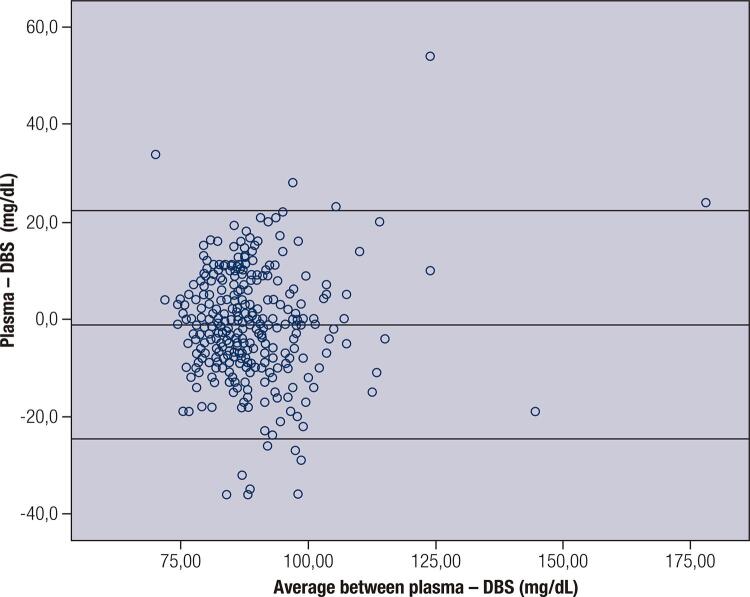
Bland-Altman plot of glucose showing relative difference between the DBS and venous methods. DBS: dried blood sample.

The concordance graph of Altman and Bland shows that the limits of concordance range from -24.6 to 22.3 mg/dL [1.37 to 1.24 mmol/L] (difference of ± 2SD). A fixed relevant bias is not observed (average difference -1.16) and there is no evidence of proportional bias (r = 0.056, *p* = 0.33). There were marked differences as well and these measured differences reached up to 50 mg/dL [2.78 mmol/L] ** (**Figure 1).

**Figure 2:**
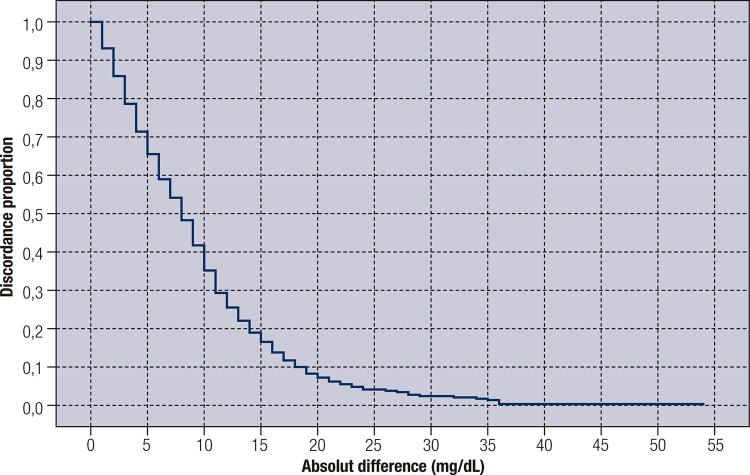
Survival-agreement plot for plasma and DBS glucose (mg/dL).

The correlation – survival-concordance plot shows that for a difference of 5 mg/dL [0.28 mmol/L] between plasma and DBS, there was a concordance of less than 40% in all samples. For a difference of 15 mg/dL [0.83 mmol/L], 85% of samples are consistent. The concordance for other tolerance limits for the difference between DBS and plasma can also be seen in Figure 2.

The Hct was available in 76.8% patients and its value ranged from 28% to 44%.

Thirty patients were anemic and 206 were non-anemic. The average glucose values in plasma and in DBS of patients with anemia were 87 ± 10.9 mg/dL [4.83 ± 0.60 mmol/L], and 90.5 ± 9.7 mg/dL [5.02 ± 0.54 mmol/L], respectively, for non-anemic patients. Their average glucose values were 87.5 ± 13 mg/dL [4.86 ± 0.72 mmol/L], and 88.8 ± 12.3 mg/dL [4.93 ± 0.68 mmol/L], respectively, with no significant difference (*p *= 0.149) (Table 2).

**Table 2:** Interference of hematocrit in blood glucose tests on filter paper

	Anemic		Non anemic	

Descriptive statistics	Glucose on the plasma	Glucose on the filter paper	Glucose on the plasma	Glucose on the filter paper
N	30	30	206	206
Average	87	90.5	87.5	88.8
Standard deviation	10.9	9.7	13	12.3
P-value of p	**0.031**		**0.149**	
ICC	**0.632**		**0.51**	

World Health Organization (2001).

ICC: interclass correlation coefficient.

In order to evaluate the storage effect and its impact on the stability of the samples in DBS, stability studies for DBS were performed in this work. The blood drops in DBS were left to dry at room temperature for approximately four hours. After that, they were placed in plastic bags and stored in a refrigerator. The samples were analyzed in periods ranging from 6 to 71 days in a total of 422 samples. The average glucose values for the samples collected in DBS did not show significant differences after storage. The average values of the samples analyzed in DBS in the initial analysis and after storage were respectively 92.27 ± 14.1 mg/dL [5.12 ± 0,78 mmol/L] and 92.51 ± 14.3 mg/dL [5.13 ± 0,79 mmol/L]. It was verified that the average values found did not present significant differences (*p *= 0.612) and the intraclass correlation coefficient ICC = 0.77 (0.73-0.81).

## DISCUSSION

The present study is the first one to evaluate the use of DBS to measure glucose levels during pregnancy. There are few studies using DBS for glucose determination measurement and none of them relates to pregnancy (10,20,26,32-35).

Ward and cols. (10) found a linear relationship between plasma glucose (r = 0.98; *p *< 0.001) and capillary glucose (r = 0.999; *p *< 0.001) in DBS in non-pregnant patients and did not find statistically significant differences between the two methods. A linear relationship between capillary glucose in DBS and plasma glucose was also observed by Abyholm (r = 0.984) (26). In the present samples from pregnant women, a linear relationship was also found (r = 0.507, *p *< 0,001).

Differences between the measurement of blood glucose in blood plasma and the one obtained from capillary blood are expected. Despite these differences, Alvarez and cols. (36) found a correlation between the methods of capillary glucose and plasma glucose in adolescents. The average of plasma glucose was 7.9 points higher than the average of blood glucose (91.5 vs. 83.6 mg/dL [5.08 vs. 4.64 mmol/L], *p *< 0.001; ICC = 0.419). Differently, in our samples from pregnant women, we did not find a fixed value that would improve the correlation between plasma glucose and DBS. The capillary glucose collected in DBS, in the present study, shows good correlation with the measurement of plasma glucose in pregnant women; however, they do not show a good concordance. The absence of a fixed value and the large dispersion of the difference compromise the quality of DBS as plasma substitute in pregnant women.

In the present work, the Altman and Bland (31) method was used. This is appropriate to assess the accuracy of a measurement as compared with the reference method and it suggests that correlation is not the same as concordance. Although former studies suggest a concordance between plasma and DBS, there was no progress in the research and development of the method. DBS sampling is rarely applied clinically. However, as diabetes incidence is increasing in the world, investment is required in different methodologies in order to reduce the costs and facilitate the diagnosis.

Lacher and cols. (37), also found a linear relationship between plasma glucose and blood glucose in DBS (r = 0.81, *p *< 0.01). The average of plasma glucose was higher than the glucose in DBS 6.34 ± 2.19 vs. 6.08 ± 2.30 mmol/L [114,2 ± 39,5) vs. 109,5 ± 41,4 mg/dL]. As our data, Altman and Bland’s graph of concordance from Lacher’s study showed a relative difference between capillary and plasma glucose that may divert from -37.7 mg/dL to 26.9 mg/dL, [-2.09 to 1.49 mmol/L] however, a fixed bias was observed (-5.4% average difference).

In the present study, the glucose measured in the capillary blood in DBS is 10 to 15% lower than plasma glucose, depending on the patient’s hematocrit. Altman and Bland’s graph of concordance suggests that the difference obtained between the values by both methods may have divergence ranging from -24.6 to 22.3 mg/dL [-1.37 to 1.24 mmol/L]. In addition, a relevant fixed bias was not observed (-1.16 average difference) and there is no evidence of proportional bias (r = 0.056, *p *= 0.33). These findings make the values of the fast samples not useful for the diagnosis of gestational diabetes.

Abyholm (26) observed that there is a significant difference in the glucose concentration measured in small and big blood drops but did not find any difference between punched discs from the center or from the periphery of samples. The average recovered measurement was 98.3% ± 9.6%, r = 0.984 (n = 244). In our study, the qualification techniques required to minimize the pre-analytical variation was observed. A proper drop was obtained and used discs were removed from the sample center.

A sensibility of 51.4% and 74.7% specificity for DBS was observed when the established cut-off point was ≥ 92 mg /dL [5.11 mmol/L] and a 95.7% sensibility was found when the glucose cut-off point in DBS was reduced to 80 mg /dL [4.44 mmol/L]. However, it was found that 4.3% of the samples were comprised of false-negative results. It means that these patients would be incorrectly classified as having normal blood glucose when already presenting changes in blood glucose levels. It was further observed that a substantial increase in the frequency of false-positive results occurred, which ranges from 51 (25.2%) to 161 (79.7%). This increase of false-positive results in the samples does not reflect a problem for a screening method, considering that the patients under suspicion will undergo a new collection for analysis by the reference method.

Women with low hematocrit showed a statistically significant difference between plasma glucose and glucose dosage in DBS. Anemia falsely increased glucose levels in DBS, in agreement with other studies performed with importable instruments performed by the Center for Devices and Radiological Health (25).

There is a correlation between the values of capillary glucose measured in DBS and plasma glucose in pregnant women. Both methods do not present a good concordance, which limits their use as a tool for the diagnosis of gestational diabetes.
